# Evaluating the performance of copula models in phase I-II clinical trials under model misspecification

**DOI:** 10.1186/1471-2288-14-51

**Published:** 2014-04-14

**Authors:** Kristen Cunanan, Joseph S Koopmeiners

**Affiliations:** 1 Division of Biostatistics, School of Public Health, University of Minnesota, A460 Mayo Building, MMC 303, 420 Delaware St. SE, 55455 Minneapolis, MN, USA

**Keywords:** Bayesian Adaptive Design, Phase I-II, Copula models

## Abstract

**Background:**

Traditionally, phase I oncology trials are designed to determine the maximum tolerated dose (MTD), defined as the highest dose with an acceptable probability of dose limiting toxicities(DLT), of a new treatment via a dose escalation study. An alternate approach is to jointly model toxicity and efficacy and allow dose escalation to depend on a pre-specified efficacy/toxicity tradeoff in a phase I-II design. Several phase I-II trial designs have been discussed in the literature; while these model-based designs are attractive in their performance, they are potentially vulnerable to model misspecification.

**Methods:**

Phase I-II designs often rely on copula models to specify the joint distribution of toxicity and efficacy, which include an additional correlation parameter that can be difficult to estimate. We compare and contrast three models for the joint probability of toxicity and efficacy, including two copula models that have been proposed for use in phase I-II clinical trials and a simple model that assumes the two outcomes are independent. We evaluate the performance of the various models through simulation both when the models are correct and under model misspecification.

**Results:**

Both models exhibited similar performance, as measured by the probability of correctly identifying the optimal dose and the number of subjects treated at the optimal dose, regardless of whether the data were generated from the correct or incorrect copula, even when there is substantial correlation between the two outcomes. Similar results were observed for a simple model that assumes independence, even in the presence of strong correlation. Further simulation results indicate that estimating the correlation parameter in copula models is difficult with the sample sizes used in Phase I-II clinical trials.

**Conclusions:**

Our simulation results indicate that the operating characteristics of phase I-II clinical trials are robust to misspecification of the copula model but that a simple model that assumes independence performs just as well due to difficulty in estimating the copula model correlation parameters from binary data.

## Background

Phase I oncology trials are primarily concerned with establishing the safety profile of a new treatment via determining the maximum tolerated dose (MTD), defined as the highest dose with the probability of toxicity less than a pre-specified target toxicity rate. Dose escalation studies can be categorized as either rule-based, such as the traditional 3 + 3 [1], or model-based, such as the continual reassessment method (CRM) [2]. Standard rule-based designs are advantageous with their simplicity in implementation; however, such designs are less desirable in their performance and efficiency, since the selection probability for the true MTD can be poor and dose assignment is based on information from the current dose level only. The standard CRM uses a simple parametric model, such as a one-parameter power model or two-parameter logistic regression mode, to characterize the relationship between dose level and the probability of experiencing a dose limiting toxicity (DLT). This method assumes a monotonic dose-response relationship between dose level and toxicity and has a variety of proposed modifications to better ensure patient safety [3].

Standard phase I designs assume that both the probabilities of toxicity and efficacy of a new drug increase as dose level increases. Nevertheless, in some instances an increase in dose level may result in a substantial increase in toxicity but only a small increase in efficacy. Thus, an alternate approach is to consider the tradeoff between toxicity and efficacy during dose escalation. To this end, several phase I-II study designs that jointly model toxicity and efficacy have been discussed in the literature [4-6]. As with the CRM, these methods assume a parametric model for both the dose-toxicity and dose-efficacy relationship, which may also include a quadratic term for efficacy to allow for model flexibility. In addition, these methods require that we specify a joint probability model for efficacy and toxicity, which is often accomplished using a *copula* model.

Copula models provide a flexible framework for specifying the joint distribution of two random variables [7]. In a copula model, a joint distribution is specified on the unit square and a joint distribution for any two random variables can be derived using an inverse transformation. In the context of a phase I-II clinical trial, we specify parametric models for the dose-response relationship for toxicity and efficacy and a copula model is used to specify a joint model for efficacy and toxicity.

Model-based designs tend to surpass rule-based designs in their ability to correctly identify the MTD and in the number of patients treated at the MTD [8], but are potentially vulnerable to model misspecification. This problem is exacerbated by the presence of a copula model in efficacy/toxicity tradeoff designs. Copula models impose a rigid structure on the relationship between toxicity and efficacy, which may not accurately reflect the underlying data generating process. An incorrectly specified model could potentially lead to a decreased probability of correctly identifying the MTD and decreased number of patients treated at the MTD.

In this manuscript, we use simulation to investigate the impact of model misspecification in phase I-II clinical trials. We consider five scenarios for the true probabilities of toxicity and efficacy. Data are simulated assuming one of two copula models and fit using the correct copula, an incorrect copula, and a model that assumes independence. Data are also simulated assuming differing degrees of positive correlation between toxicity and efficacy. Our results show that the two models are relatively robust to model misspecification but that the independence model actually performs better in many cases.

The remaining sections of this manuscript are organized as follows. In Section ‘Methods’, we introduce several joint probability models used in phase I-II clinical trials and describe the dose-finding algorithm used in our simulation study. In Section ‘Results and discussion’, we present simulation results evaluating the performance of the two copula models when correctly specified and under model specification. Finally, we conclude with a brief discussion in Section ‘Conclusions’.

## Methods

In this section, we introduce two joint probability models used in phase I-II clinical trials. In both cases, we specify marginal models for the probabilities of toxicity and efficacy and develop a joint model using a copula model. First, we specify models for the marginal probability of toxicity and the marginal probability of efficacy.

Let *Y*_*T*_ and *Y*_*E*_ be the binary indicators of toxicity and efficacy, respectively. Denote *π*(*y*_*T*_,*y*_*E*_|*z*)=*Pr*(*Y*_*T*_=*y*_*T*_,*Y*_*E*_=*y*_*E*_|*z*) as the joint probability of toxicity and efficacy given dose level *z*, with marginal probabilities of toxicity and efficacy, *π*_*T*_ and *π*_*E*_, respectively, also functions of *z*. We can model the dose-toxicity and dose-efficacy relationships with any monotonic function. For simplicity, we assume logistic regression models for efficacy and toxicity as follows:

(1) $$\log\left(\frac{\pi_{T}}{1-\pi_{T}}\right)=\beta_{0,T}+\beta_{1,T}(z-1),$$

(2) $$\text{and}\;\log\left(\frac{\pi_{E}}{1-\pi_{E}}\right)=\beta_{0,E}+\beta_{1,E}(z-1)+\beta_{2,E}{\left(z-1\right)}^{2}.$$

We include a quadratic term for efficacy to allow model flexibility should the probability of efficacy level off or diminish after a certain dose level. We note that the intercept terms in (1) and (2) correspond to the log-odds of toxicity and efficacy, respectively, at the first dose level. This is useful for interpretation and prior specification purposes. We next describe two copula models used in phase I-II clinical trials for specifying a joint distribution for *Y*_*T*_ and *Y*_*E*_.

### Braun copula

We first consider the copula model discussed by Arnold and Strauss [9] and applied to joint modeling of efficacy and toxicity in the setting of a Phase I-II clinical trial by Braun [5]. These authors specify the joint distribution of *Y*_*T*_ and *Y*_*E*_ as:

(3) $$\begin{array}{ll} \pi (y_{T},y_{E}|z)= & k(\pi_{T},\pi_{E},\psi_{1})\pi_{E}^{y_{E}}{\left(1-\pi_{E}\right)}^{1-y_{E}}\pi_{T}^{y_{T}}\\ & \times {\left(1-\pi_{T}\right)}^{1-y_{T}}\psi_{1}^{y_{T}y_{E}}{\left(1-\psi_{1}\right)}^{1-y_{T}y_{E}}. \end{array}$$

Here, *ψ*_1_ represents the correlation between *Y*_*T*_ and *Y*_*E*_ and takes on values between 0 and 1. *ψ*_1_ greater than 0.5 reflects positive correlation, *ψ*_1_ less than 0.5 reflects negative correlation and *ψ*_1_=0.5 represents independence. We note also that *k*(*π*_*T*_,*π*_*E*_,*ψ*_1_) is a constant that is included to assure that the four probabilities sum to 1 and depends on *π*_*T*_, *π*_*E*_, and *ψ*_1_. The conditional probability of *Y*_*E*_|*Y*_*T*_ can be derived from the joint probability in (3) and is equal to:

$$\pi_{E|T}=\frac{\pi_{E}\psi_{1}^{y_{T}}{\left(1-\psi_{1}\right)}^{1-y_{T}}}{\pi_{E}\psi_{1}^{y_{T}}{\left(1-\psi_{1}\right)}^{1-y_{T}}+(1-\psi_{1})(1-\pi_{E})}.$$

An analogous conditional probability of *Y*_*T*_|*Y*_*E*_ can also be derived.

There are two key properties of the above model that are worthy of discussion. First, the correlation parameter, *ψ*_1_, has the useful interpretation that *ψ*_1_/(1−*ψ*_1_) is the odds ratio between *Y*_*E*_ and *Y*_*T*_. A second, less desirable property, is that *π*_*T*_ and *π*_*E*_ are no longer the marginal probabilities of *Y*_*T*_ and *Y*_*E*_ equal to 1, respectively, if *ψ*_1_≠.5. Instead, the marginal probability of *Y*_*E*_ equal to 1 is:

$$\begin{array}{ll} \text{Pr}(Y_{E}=1)= & k(\pi_{T},\pi_{E},\psi_{1})\pi_{E}\left(\left(1-\pi_{T}\right)\left(1-\psi_{1}\right)\right)\\ & \left(+\;\pi_{T}\psi_{1}\right) \end{array}$$

and the marginal probability of *Y*_*T*_ equal to 1 is:

$$\begin{array}{ll} \text{Pr}(Y_{T}=1)= & k(\pi_{T},\pi_{E},\psi_{1})\pi_{T}\left(\left(1-\pi_{E}\right)\left(1-\psi_{1}\right)\right)\\ & \left(+\;\pi_{E}\psi_{1}\right). \end{array}$$

This is a key point that must be considered during dose finding.

### Gumbel copula

Thall and Cook [4] instead model the joint probability of efficacy and toxicity using the Gumbel copula discussed by Murtaugh and Fisher [10], which implies the following joint probability model for *Y*_*T*_ and *Y*_*E*_:

(4) $$\begin{array}{ll} \pi (y_{T},y_{E}|z)= & \pi_{E}^{y_{E}}{\left(1-\pi_{E}\right)}^{1-y_{E}}\pi_{T}^{y_{T}}{\left(1-\pi_{T}\right)}^{1-y_{T}}\\ & +{\left(-1\right)}^{y_{E}+y_{T}}\pi_{E}(1-\pi_{E})\pi_{T}(1-\pi_{T})\psi_{2}. \end{array}$$

Here, *ψ*_2_∈(−1,1) captures the correlation between *Y*_*T*_ and *Y*_*E*_, with *ψ*_2_=0 implying independence and *ψ*_2_∈(0,1) implying positive correlation. We can again derive the conditional probability of *Y*_*E*_ given *Y*_*T*_,

$$\pi_{E|T}=\pi_{E}+{\left(-1\right)}^{1+y_{T}}\pi_{E}(1-\pi_{E})\pi_{T}^{1-y_{T}}{\left(1-\pi_{T}\right)}^{y_{T}}\psi_{2}.$$

The conditional probability of *Y*_*T*_ given *Y*_*E*_ can be expressed in an analogous fashion.

An advantage of this model is that both *π*_*E*_ and *π*_*T*_ retain their original interpretations as the marginal probabilities of efficacy and toxicity, respectively. This can be easily seen by summing *P*(*Y*_*E*_=1,*Y*_*T*_=1) and *P*(*Y*_*E*_=1,*Y*_*T*_=0) from (4). Unlike the Braun Copula, the correlation parameter for the Gumbel copula, *ψ*_2_, does not have a straight-forward interpretation.

### Independent model

An alternate approach would be to assume independence between *Y*_*T*_ and *Y*_*E*_, in which case the joint probability of toxicity and efficacy is simply the product of the marginal probabilities,

(5) $$\pi (y_{T},y_{E}|z)=\pi_{T}^{y_{T}}{\left(1-\pi_{T}\right)}^{1-y_{T}}\pi_{E}^{y_{E}}{\left(1-\pi_{E}\right)}^{1-y_{E}},$$

which is of course what we get by setting *ψ*_1_=0.5 and *ψ*_2_=0 in the Braun and Gumbel copulas, respectively. While it is unlikely that this model accurately reflects the true association between *Y*_*T*_ and *Y*_*E*_, this model may still be useful because the sample size in phase I-II oncology trials is limited and we may lack the sample size to precisely estimate *ψ*_1_ and *ψ*_2_. If the likelihood contains very little information about these parameters, it may be that we do not lose much with respect to our ability to identify the optimal dose by assuming independence instead of a more complicated model.

### Likelihood and priors

Let (*y*_*T*,1_,*y*_*E*,1_),(*y*_*T*,2_,*y*_*E*,2_),…,(*y*_*T*,*n*_,*y*_*E*,*n*_) be pairs of binary toxicity and efficacy outcomes at dose levels (*z*_1_,*z*_2_,…,*z*_*n*_). The full likelihood for the models described above is:

$$\begin{array}{lll} L(\vec{\beta }|\vec{y_{T}},\vec{y_{E}},\vec{z})= & \overset{n}{\underset{i=1}{\prod }}\pi {\left(1,1|z_{i}\right)}^{y_{T,i}y_{E,i}}\pi {\left(0,1|z_{i}\right)}^{\left(1-y_{T,i}\right)y_{E,i}}\; & \;\\ & \times \pi {\left(1,0|z_{i}\right)}^{y_{T,i}\left(1-y_{E,i}\right)}\pi \; & \;\\ & \times {\left(0,0|z_{i}\right)}^{\left(1-y_{T,i}\right)\left(1-y_{E,i}\right)}\; & \; \end{array}$$

where *π*(*Y*_*T*_,*Y*_*E*_|*z*) is defined using either (3), (4) or (5) and $\vec{\beta }=(\beta_{0,T},\beta_{1,T},\beta_{0,E},\beta_{1,E},\beta_{2,E},\psi_{k})$ with *k*=1,2 for the two copula models and $\vec{\beta }=(\beta_{0,T},\beta_{1,T},\beta_{0,E},\beta_{1,E},\beta_{2,E})$ for the independence model.

We must specify a prior distribution for each regression and association parameter, to complete a Bayesian analysis. We specify the following normal priors for the two intercept terms and the quadratic term for efficacy: *β*_0,*T*_∼*N*(−3,*sd*=3), *β*_0,*E*_∼*N*(−1,3), and $\beta_{2,E}∼N\left(0,\frac{1}{4}\right)$. The priors for *β*_0,*T*_ and *β*_0,*E*_ correspond to a prior belief of *P*(*Y*_*T*_=1|*z*=1)=0.05 and *P*(*Y*_*E*_=1|*z*=1)=0.27 but provide sufficient support over all plausible values for *β*_0,*T*_ and *β*_0,*E*_ and represent only mildly informative priors. The prior for *β*_2,*E*_ is chosen to reflect a strong belief against a quadratic relationship but allows the model flexibility should there be drastic departures from a linear relationship. Gamma priors were set for *β*_1,*T*_ and *β*_1,*E*_ with mean 1 and standard deviation 2, corresponding to a $\text{Gamma}(\frac{1}{4},\frac{1}{4})$. Assuming Gamma priors for *β*_1,*T*_ and *β*_1,*E*_ implies that the marginal probability of toxicity will be monotonically increasing but the same is not true for the marginal probability of efficacy due to the inclusion of a quadratic term for the marginal probability of efficacy. Finally, we specify non-informative uniform priors for the association parameters: *Uniform*(0,1) for *ψ*_1_ and *Uniform*(−1,1) for *ψ*_2_.

### Dose-finding algorithm

For our simulation study, we follow the dose-finding algorithm proposed by Thall and Cook [4]. These authors identify a set of acceptable doses by defining a maximum acceptable probability of toxicity assuming 100% efficacy, a minimum acceptable probability of efficacy assuming no toxicity and define a desirability index to identify the optimal dose from the set of acceptable doses.

Let ${\bar{\pi }}_{T}$ be the maximum acceptable probability of toxicity assuming 100% efficacy and ${\underline{\pi }}_{E}$ be the minimum acceptable probability of efficacy assuming no toxicity, as specified by the physician. A dose, *z*, is acceptable if the posterior probabilities of the two events $\pi_{T}(z)<{\bar{\pi }}_{T}$ and $\pi_{E}(z)>{\underline{\pi }}_{E}$ exceed a pre-specified threshold, *p*, i.e.

(6) $$\text{Pr}(\pi_{T}(z)<{\bar{\pi }}_{T},\pi_{E}(z)>{\underline{\pi }}_{E}|\text{Data},z)>\mathrm{p.}$$

The trial terminates for futility if, at any point during the trial, all doses are unacceptable according to Equation (6). The optimal dose is selected from the set of acceptable doses using a desirability index. The desirability index for a (*π*_*T*_(*z*),*π*_*E*_(*z*)) pair is defined by Thall and Cook as follows:

(7) $$D(z)=1-{\left({\left(\frac{\pi_{T}(z)}{{\bar{\pi }}_{T}}\right)}^{q}+{\left(\frac{1-\pi_{E}(z)}{1-{\underline{\pi }}_{E}}\right)}^{q}\right)}^{1/q},$$

where *q* is defined by identifying a probability of toxicity and probability of efficacy pair, $\left(\pi_{T}^{*},\pi_{E}^{*}\right)$, that is equally desirable to $\left({\bar{\pi }}_{T},1.0\right)$ and $\left(0,{\underline{\pi }}_{E}\right)$, plugging $\left(\pi_{T}^{*},\pi_{E}^{*}\right)$ into (7) and solving for *q* when *D*(*z*) equals 0. Larger values of *D*(*z*) are considered more desirable and the optimal combination, (0.0,1.0), has *D*(*z*) equal to 1 regardless of *q*.

The dose-finding algorithm proceeds as follows: 

1. Treat the first cohort of *m* patients at the lowest dose level.

2. Update the posterior distributions of the probabilities of toxicity and efficacy for each dose level using data from all previous cohorts.

3. Identify the set of acceptable doses using criterion (6). If no dose is found acceptable, terminate for futility.

4. Treat the next cohort at the dose that maximizes *D*(*z*) under the restriction that dose levels may not be skipped when escalating. Return to step 2.

5. Repeat steps 2–4 until the maximum sample size is reached. The dose that maximizes *D*(*z*) at study completion is considered the optimal dose.

## Results and discussion

We completed a small simulation study to evaluate the performance of phase I-II clinical trials when the copula model is misspecified. Trial parameters were set as follows. We assume a cohort size of 3 patients with a maximum of 15 cohorts, for a maximum sample size of 45 patients. The maximum acceptable probability of toxicity assuming 100% efficacy and minimum acceptable probability of efficacy assuming no toxicity were set at ${\underline{\pi }}_{T}=0.5$ and ${\bar{\pi }}_{E}=0.55$, respectively. We set $\left(\pi_{T}^{*},\pi_{E}^{*}\right)$ equal to (0.25,0.60), which corresponds to *q*=2. The pre-specified threshold, *p*, to determine the set of acceptable doses using the posterior probability of toxicity and efficacy is assumed to be 0.05. We consider four dose levels with dose index, *z*={1,2,3,4}. All simulations were completed in R version 2.15.1 [11]. Gibbs sampling was completed in JAGS as called from R using rjags [12]. We simulate 1000 trials; within each trial, 1000 iterations were kept for inference following a period of 5000 iterations for burn-in.

### Scenarios

We simulated data from five scenarios to evaluate the performance of our models in a variety of settings. Table 1 contains the true marginal probability of toxicity, marginal probability of efficacy and *D*(*z*) at each dose level for the five scenarios, with the optimal dose in bold. Scenarios 1–3 represent the case where the probability of toxicity and efficacy increase as dose increases with the optimal dose ranging from dose level 1 to dose level 4. In scenario 4, both dose levels three and four are acceptable but dose level three is the optimal dose. Finally, in scenario 5, all doses are safe but none have acceptable efficacy and the correct decision is to terminate for futility.

**Table 1:** **True marginal probability of toxicity, marginal probability of efficacy and *D *** **(*z*) for each dose level**

**Scenario**				**Dose**	
		**1**	**2**	**3**	**4**
1	P(Toxicity)	0.05	0.12	**0.27**	0.50
	P(Efficacy)	0.38	0.55	**0.71**	0.83
	D(z)	-0.38	-0.03	**0.16**	-0.07
2	P(Toxicity)	**0.38**	0.52	0.67	0.79
	P(Efficacy)	**0.77**	0.82	0.86	0.89
	D(z)	**0.08**	-0.11	-0.38	-0.6
3	P(Toxicity)	0.02	0.07	0.15	**0.31**
	P(Efficacy)	0.12	0.25	0.45	**0.67**
	D(z)	-0.96	-0.67	-0.26	**0.04**
4	P(Toxicity)	0.05	0.11	**0.25**	0.46
	P(Efficacy)	0.18	0.55	**0.79**	0.86
	D(z)	-0.82	-0.02	**0.32**	0.03
5	P(Toxicity)	0.03	0.08	0.18	0.38
	P(Efficacy)	0.18	0.25	0.33	0.43
	D(z)	-0.82	-0.67	-0.53	-0.48

Optimal dose is in bold.

For each scenario, we simulated data from both copula models and fit the data using either the correct copula model, the incorrect copula model or the independence model. The association parameters, *ψ*_1_ and *ψ*_2_, were varied to determine the impact of the correlation of *Y*_*T*_ and *Y*_*E*_ on model performance under model misspecification. The Braun association parameter, *ψ*_1_, in (3) ranges from 0.5 to 0.9 by increments of 0.2; this corresponds to an odds ratio between toxicity and efficacy ranging from 1 to 9. The Gumbel association parameter, *ψ*_2_ in (4) ranges from 0 to 0.8 by increments of 0.4. Recall that efficacy and toxicity are independent if *ψ*_1_ equals 0.5 for the Braun model and *ψ*_2_ equals 0 for the Gumbel model. In these cases, the independence model is the correct model and the Braun and Gumbel models are unnecessarily trying to estimate a correlation parameter when the two endpoints are actually independent.

### Results

Tables 2, 3, 4, 5 and 6 display the performance of the Braun, Gumbel, and independence models for the five scenarios at no, moderate, and very strong degrees of positive association; each table summarizes the results for data simulated from both the Braun and Gumbel copula models. Within a model stratum, the first row displays the posterior probability of selecting that dose; while the second row displays the average number of patients treated at that dose. Operating characteristics for the optimal dose are in bold.

**Table 2:** Results using the Braun and Gumbel copulas for data simulation under Scenario 1

**Data**	***ψ***_***k***_	**Model**			**Dose**		
			**Futility**	**1**	**2**	**3**	**4**
Braun	0.5	Braun	0.039	0.045	0.212	**0.475**	0.229
				5.91	12.792	**17.13**	8.298
		Gumbel	0.038	0.039	0.225	**0.482**	0.216
				6.075	12.738	**16.596**	8.775
		Indep	0.037	0.046	0.223	**0.504**	0.19
				6	13.146	**16.923**	8.115
	0.7	Braun	0.037	0.034	0.197	**0.514**	0.218
				5.667	12.966	**17.496**	7.86
		Gumbel	0.023	0.032	0.235	**0.524**	0.186
				5.613	13.32	**17.562**	7.941
		Indep	0.033	0.035	0.219	**0.528**	0.185
				5.796	13.356	**17.334**	7.77
	0.9	Braun	0.024	0.018	0.208	**0.514**	0.236
				5.19	12.888	**17.58**	8.814
		Gumbel	0.011	0.037	0.225	**0.538**	0.189
				5.901	13.428	**17.895**	7.542
		Indep	0.006	0.041	0.226	**0.526**	0.201
				5.994	13.605	**17.349**	7.95
Gumbel	0	Braun	0.042	0.033	0.217	**0.503**	0.205
				6.054	12.786	**17.007**	8.373
		Gumbel	0.042	0.041	0.21	**0.499**	0.208
				6.033	12.651	**17.094**	8.28
		Indep	0.034	0.054	0.208	**0.478**	0.226
				6.591	12.447	**16.671**	8.622
	0.4	Braun	0.036	0.034	0.216	**0.506**	0.208
				5.871	12.828	**17.157**	8.322
		Gumbel	0.029	0.039	0.222	**0.461**	0.249
				5.775	13.287	**16.029**	9.081
		Indep	0.018	0.044	0.213	**0.501**	0.224
				5.946	12.81	**17.205**	8.67
	0.8	Braun	0.036	0.028	0.237	**0.475**	0.224
				5.886	13.875	**16.119**	8.307
		Gumbel	0.016	0.036	0.231	**0.506**	0.211
				5.877	13.182	**17.34**	8.22
		Indep	0.032	0.032	0.216	**0.524**	0.196
				5.388	13.242	**17.616**	8.079

The first row is the selection probability for dose *z*; and the second is the average number of patients treated at dose *z*. *Y*_*T*_ and *Y*_*S*_ are simulated with association, *ψ*_*k*_; *k*=1,2 when appropriate. The operating characteristics for the target dose are in bold.

**Table 3:** Results using the Braun and Gumbel copulas for data simulation under Scenario 2

**Data**	***ψ***_***k***_	**Model**			**Dose**		
			**Futility**	**1**	**2**	**3**	**4**
Braun	0.5	Braun	0.147	**0.748**	0.094	0.011	0
				**33.159**	5.922	0.798	0.042
		Gumbel	0.13	**0.732**	0.121	0.016	0.001
				**32.652**	6.984	1.161	0.084
		Indep	0.129	**0.770**	0.093	0.008	0
				**34.542**	5.826	0.825	0.063
	0.7	Braun	0.16	**0.744**	0.091	0.005	0
				**32.919**	6.315	0.645	0.039
		Gumbel	0.106	**0.761**	0.117	0.016	0
				**34.059**	6.762	0.81	0.09
		Indep	0.098	**0.781**	0.109	0.012	0
				**34.671**	6.516	0.705	0.09
	0.9	Braun	0.204	**0.707**	0.086	0.003	0
				**31.878**	6.18	0.396	0.018
		Gumbel	0.071	**0.799**	0.114	0.015	0.001
				**34.977**	7.2	0.63	0.063
		Indep	0.095	**0.773**	0.122	0.009	0.001
				**34.329**	6.789	0.795	0.078
Gumbel	0	Braun	0.14	**0.722**	0.126	0.012	0
				**32.889**	6.69	0.756	0.051
		Gumbel	0.124	**0.745**	0.122	0.008	0.001
				**33.714**	6.564	0.786	0.069
		Indep	0.13	**0.751**	0.111	0.007	0.001
				**33.585**	6.465	0.738	0.099
	0.4	Braun	0.159	**0.741**	0.09	0.009	0.001
				**33.39**	5.886	0.96	0.066
		Gumbel	0.127	0.**755**	0.1	0.016	0.002
				**33.882**	6.234	1.02	0.09
		Indep	0.118	**0.758**	0.113	0.011	0
				**33.36**	7.059	0.927	0.036
	0.8	Braun	0.16	**0.742**	0.094	0.004	0
				**32.991**	6.048	0.534	0.06
		Gumbel	0.081	**0.799**	0.104	0.014	0.002
				**35.247**	6.657	0.84	0.048
		Indep	0.113	**0.76**	0.118	0.009	0
				**34.068**	6.528	0.801	0.081

The first row is the selection probability for dose *z*; and the second is the average number of patients treated at dose *z*. *Y*_*T*_ and *Y*_*S*_ are simulated with association, *ψ*_*k*_; *k*=1,2 when appropriate. The operating characteristics for the target dose are in bold.

**Table 4:** Results using the Braun and Gumbel copulas for data simulation under Scenario 3

**Data**	***ψ***_***k***_	**Model**			**Dose**		
			**Futility**	**1**	**2**	**3**	**4**
Braun	0	Braun	0.195	0.001	0.004	0.045	**0.755**
				3.426	3.636	5.358	**27.504**
		Gumbel	0.174	0.001	0.005	0.042	**0.778**
				3.459	3.744	5.418	**27.855**
		Indep	0.149	0.001	0.003	0.035	**0.812**
				3.384	3.69	4.938	**29.169**
	0.4	Braun	0.162	0.001	0.005	0.025	**0.807**
				3.297	3.762	4.884	**28.719**
		Gumbel	0.139	0	0.003	0.042	**0.816**
				3.417	3.693	5.487	**29.025**
		Indep	0.13	0	0.003	0.049	**0.818**
				3.423	3.705	5.547	**28.857**
	0.8	Braun	0.143	0	0.002	0.033	**0.822**
				3.351	3.552	5.502	**29.19**
		Gumbel	0.101	0	0.002	0.037	**0.86**
				3.387	3.636	5.568	**29.775**
		Indep	0.093	0	0.002	0.04	**0.865**
				3.381	3.804	5.628	**29.592**
Gumbel	0	Braun	0.189	0.001	0.004	0.047	**0.759**
				3.327	3.585	5.31	**27.942**
		Gumbel	0.173	0	0.004	0.044	**0.779**
				3.336	3.675	5.313	**28.224**
		Indep	0.16	0.001	0	0.034	**0.805**
				3.435	3.789	5.172	**28.434**
	0.4	Braun	0.16	0	0.001	0.048	**0.791**
				3.45	3.63	5.61	**28.101**
		Gumbel	0.163	0	0.004	0.049	**0.784**
				3.363	3.666	5.49	**28.164**
		Indep	0.156	0	0.001	0.034	**0.809**
				3.405	3.591	4.977	**28.875**
	0.8	Braun	0.171	0.001	0.001	0.029	**0.798**
				3.342	3.645	5.25	**28.116**
		Gumbel	0.156	0.001	0.004	0.029	**0.81**
				3.384	3.702	5.262	**28.776**
		Indep	0.144	0	0.002	0.042	**0.812**
				3.342	3.804	5.484	**28.824**

The first row is the selection probability for dose *z*; and the second is the average number of patients treated at dose *z*. *Y*_*T*_ and *Y*_*S*_ are simulated with association, *ψ*_*k*_; *k*=1,2 when appropriate. The operating characteristics for the target dose are in bold.

**Table 5:** Results using the Braun and Gumbel copulas for data simulation under Scenario 4

**Data**	***ψ***_***k***_	**Model**			**Dose**		
			**Futility**	**1**	**2**	**3**	**4**
Braun	0.5	Braun	0.023	0.003	0.057	**0.665**	0.252
				3.42	6.39	**23.19**	11.268
		Gumbel	0.015	0.001	0.055	**0.683**	0.246
				3.402	7.041	**23.358**	10.809
		Indep	0.017	0.003	0.078	**0.621**	0.281
				3.351	7.071	**22.74**	11.304
	0.7	Braun	0.02	0.001	0.057	**0.684**	0.238
				3.333	6.849	**24.066**	10.146
		Gumbel	0.019	0.003	0.069	**0.671**	0.238
				3.39	7.107	**23.286**	10.68
		Indep	0.01	0.001	0.085	**0.655**	0.249
				3.39	7.593	**22.854**	10.875
	0.9	Braun	0.017	0.001	0.062	**0.658**	0.262
				3.303	6.996	**23.376**	10.839
		Gumbel	0.002	0	0.078	**0.698**	0.222
				3.315	7.476	**23.46**	10.695
		Indep	0.007	0.001	0.081	**0.647**	0.264
				3.543	7.743	**22.329**	11.172
Gumbel	0	Braun	0.027	0.002	0.064	**0.664**	0.243
				3.282	6.885	**23.109**	11.034
		Gumbel	0.027	0.001	0.065	**0.657**	0.25
				3.351	6.711	**22.95**	11.136
		Indep	0.025	0.002	0.084	**0.633**	0.256
				3.375	7.374	**22.767**	10.788
	0.4	Braun	0.025	0.002	0.066	**0.631**	0.276
				3.504	7.338	**22.515**	11.013
		Gumbel	0.021	0.002	0.074	**0.655**	0.248
				3.45	7.296	**22.599**	11.013
		Indep	0.013	0.001	0.069	**0.649**	0.268
				3.315	7.032	**22.542**	11.787
	0.8	Braun	0.012	0.002	0.063	**0.65**	0.273
				3.318	7.152	**22.647**	11.502
		Gumbel	0.019	0.001	0.075	**0.669**	0.236
				3.297	7.038	**23.154**	10.923
		Indep	0.013	0.004	0.075	**0.673**	0.235
				3.33	7.389	**23.172**	10.755

The first row is the selection probability for dose *z*; and the second is the average number of patients treated at dose *z*. *Y*_*T*_ and *Y*_*S*_ are simulated with association, *ψ*_*k*_; *k*=1,2 when appropriate. The operating characteristics for the target dose are in bold.

**Table 6:** Results using the Braun and Gumbel copulas for data simulation under Scenario 5

**Data**	***ψ***_***k***_	**Model**			**Dose**		
			**Futility**	**1**	**2**	**3**	**4**
Braun	0.5	Braun	**0.87**	0.001	0.005	0.023	0.101
				4.086	4.59	4.62	11.946
		Gumbel	**0.872**	0	0.006	0.022	0.1
				4.05	4.44	4.896	11.316
		Indep	**0.872**	0.002	0.009	0.016	0.101
				4.176	4.656	4.803	11.319
	0.7	Braun	**0.928**	0	0.004	0.009	0.059
				4.065	4.209	4.56	10.095
		Gumbel	**0.887**	0.002	0.014	0.014	0.083
				3.936	4.485	4.869	11.748
		Indep	**0.895**	0.002	0.01	0.016	0.077
				4.254	4.467	4.596	11.256
	0.9	Braun	**0.945**	0.001	0.004	0.009	0.041
				4.053	4.29	4.566	9.531
		Gumbel	**0.904**	0.003	0.006	0.016	0.071
				4.248	4.701	5.148	11.091
		Indep	**0.905**	0	0.006	0.016	0.073
				4.227	4.608	5.196	11.22
Gumbel	0	Braun	**0.895**	0.001	0.011	0.01	0.083
				4.035	4.41	4.674	11.196
		Gumbel	**0.878**	0.003	0.008	0.015	0.096
				3.999	4.293	4.692	11.391
		Indep	**0.897**	0.001	0.007	0.014	0.081
				4.197	4.494	4.659	11.886
	0.4	Braun	**0.898**	0.002	0.01	0.013	0.077
				3.966	4.254	4.368	11.061
		Gumbel	**0.901**	0.001	0.004	0.021	0.073
				4.179	4.452	4.89	11.112
		Indep	**0.892**	0.004	0.006	0.018	0.08
				4.065	4.365	4.644	11.559
	0.8	Braun	**0.913**	0.003	0.005	0.013	0.066
				4.002	4.536	4.623	10.794
		Gumbel	**0.925**	0.002	0.005	0.013	0.055
				4.152	4.488	4.632	11.028
		Indep	**0.897**	0.003	0.006	0.012	0.082
				4.179	4.611	4.452	11.286

The first row is the selection probability for dose *z*; and the second is the average number of patients treated at dose *z*. *Y*_*T*_ and *Y*_*S*_ are simulated with association, *ψ*_*k*_; *k*=1,2 when appropriate. The operating characteristics for the target dose are in bold.

Table 2 displays results for Scenario 1. Concentrating first on the results when data are generated from the Braun model, we see that both copula models perform similarly well in their ability to select the correct dose and in the number of patients treated at the optimal dose regardless of the true correlation. The probability of correctly identifying the optimal dose differs by less than 0.024 and the number of patients treated at the optimal dose differs by less than 1 across the three correlation conditions. Surprisingly, the independence model also performs very well regardless of the true correlation and has the highest probability of identifying the optimal dose in two of the three correlation conditions. Although, the differences are small and the independence model has essentially the same performance as the two copula models.

Similar results are observed when data are generated from the Gumbel model. Both copula models performed similarly with respect to the probability of accurately identifying the optimal dose and the number of patients treated at the optimal dose. The independence model again exhibits good performance across the three scenarios, which is surprising because the independence model lacks the flexibility to model the correlation between the two outcomes. Across the three correlation scenarios, the probability of correctly identifying the optimal dose differed by less than 0.05 and the average number of patients treated at the optimal dose differed by less than 1.5 between the three models.

Results for Scenarios 2 and 3 are found in Tables 3 and 4, respectively. The results for Scenarios 2 and 3 are similar to the results for Scenario 1. The probability of correctly identifying the optimal dose and the average number of patients treated at the optimal dose are similar for both copula models regardless of how the data are generated and the independence model provides similar performance even though the independence model is unable to appropriately model the correlation between *Y*_*T*_ and *Y*_*E*_.

Scenario 4 (Table 5), represents the scenario where multiple dose levels are acceptable, dose levels 3 and 4, but dose level 3 is optimal. This scenario represents one of the primary motivations for phase I-II designs as there is a dose level where further escalation results in a greater probability of toxicity but relatively little efficacy benefit. The results for Scenario 4 are consistent with our previous results: there is little difference between the three models in the probability of correctly identifying the optimal dose and the average number of patients treated at the optimal dose regardless of the correlation between endpoints and how the data are generated. Finally, the results for Scenario 5 can be found in Table 6. In this scenario, all dose levels are safe but have unacceptable efficacy and the correct decision is to terminate for futility. The Gumbel and independence models exhibit similar performance across all scenarios but we do observe that the Braun model is more likely to terminate for futility when *Y*_*T*_ and *Y*_*E*_ are correlated and the data are generated for the Braun model. Although, the differences are small in both cases.

**Figure 1:**
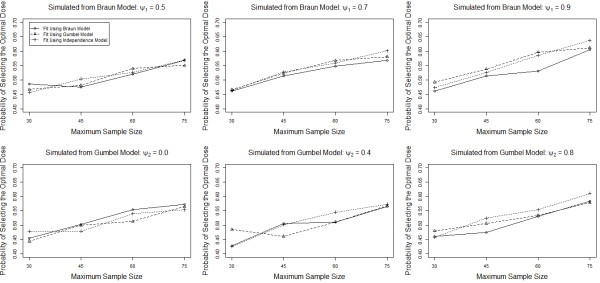
Probability of correctly identifying the optimal dose for Scenario 1 for the Braun, Gumbel and Independence model for various combinations of the true model, level of correlation and maximum sample size.

The simulations results presented in Tables 2, 3, 4, 5 and 6 indicate that specifying the correct copula model has little impact on the operating characteristics of Phase I-II clinical trials but are dependent on a number of factors, including the sample size and priors specified for the logistic regression models used for efficacy and toxicity. In order to account for these factors, we completed additional simulations to evaluate the robustness of our conclusions to changing the sample size and prior distributions. Figure 1 presents simulation results to evaluate the impact of sample size on our conclusions. Presented are the simulated probabilities of correctly identifying the optimal dose for Scenario 1 for maximum sample sizes of 30, 45, 60 and 75 subjects, with data simulated from both models and various levels of correlation. As expected, the probability of correctly identifying the optimal dose increases as the maximum sample size increases. More importantly, increasing the sample size appears to have no impact on our primary conclusion that incorrectly specifying the copula model has little impact on the probability of correctly identifying the optimal dose regardless of the level of correlation between the two endpoints and the model from which the data are generated.

**Figure 2:**
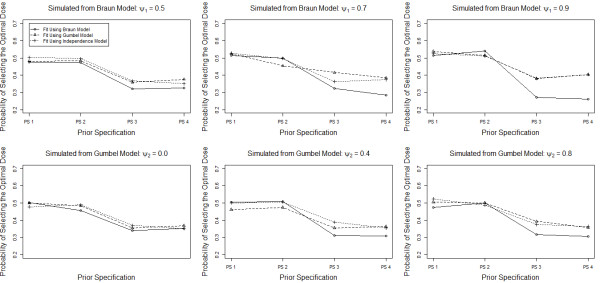
**Probability of correctly identifying the optimal dose for Scenario 1 for the Braun, Gumbel and Independence model for various combinations of the true model, level of correlation and prior specification for the regression parameters in the logistic regression models for efficacy and toxicity.** PS 1: *β*_0,*T*_∼*N*(−3,3), *β*_0,*E*_∼*N*(−1,3), $\beta_{1,T}∼\text{Gamma}\left(\frac{1}{4},\frac{1}{4}\right)$, $\beta_{1,E}∼\text{Gamma}\left(\frac{1}{4},\frac{1}{4}\right)$; PS 2: *β*_0,*T*_∼*N*(−3,5), *β*_0,*E*_∼*N*(−1,5), $\beta_{1,T}∼\text{Gamma}\left(\frac{1}{4},\frac{1}{4}\right)$, $\beta_{1,E}∼\text{Gamma}\left(\frac{1}{4},\frac{1}{4}\right)$; PS 3: *β*_0,*T*_∼*N*(−3,3), *β*_0,*E*_∼*N*(−1,3), $\beta_{1,T}∼\text{Gamma}\left(\frac{1}{25},\frac{1}{25}\right)$, $\beta_{1,E}∼\text{Gamma}\left(\frac{1}{25},\frac{1}{25}\right)$; PS 4: *β*_0,*T*_∼*N*(−3,5), *β*_0,*E*_∼*N*(−1,5), $\beta_{1,T}∼\text{Gamma}\left(\frac{1}{25},\frac{1}{25}\right)$, $\beta_{1,E}∼\text{Gamma}\left(\frac{1}{25},\frac{1}{25}\right)$.

Figure 2 presents simulation results to evaluate the impact of changing the priors for the parameters of the logistic regression models for efficacy and toxicity. We considered four prior specifications. Prior specification 1 is the original priors: *β*_0,*T*_∼*N*(−3,*sd*=3), *β*_0,*E*_∼*N*(−1,3), $\beta_{1,T}∼\text{Gamma}\left(\frac{1}{4},\frac{1}{4}\right)$ and $\beta_{1,E}∼\text{Gamma}\left(\frac{1}{4},\frac{1}{4}\right)$, in prior specification 2, we increase the prior variances for the intercept parameters: *β*_0,*T*_∼*N*(−3,5) and *β*_0,*E*_∼*N*(−1,5), in prior specification 3, we increase the prior variances for the slope parameters but not the intercept parameters: $\beta_{1,T}∼\text{Gamma}\left(\frac{1}{25},\frac{1}{25}\right)$ and $\beta_{1,E}∼\text{Gamma}\left(\frac{1}{25},\frac{1}{25}\right)$ and in prior specification 4, we increase the prior variances of both the slope and intercept parameters: *β*_0,*T*_∼*N*(−3,5), *β*_0,*E*_∼*N*(−1,5), $\beta_{1,T}∼\text{Gamma}\left(\frac{1}{25},\frac{1}{25}\right)$ and $\beta_{1,E}∼\text{Gamma}\left(\frac{1}{25},\frac{1}{25}\right)$. Presented are the simulated probabilities of correctly identifying the optimal dose for Scenario 1 for the four prior specifications, with data simulated from both models and various levels of correlation. We see that increasing the prior variance of the intercept terms has little impact on the probability of correctly identifying the optimal dose but increasing the variance of the slope decreases the probability of correctly identifying the optimal dose considerably. More importantly, we see that the independence model performs as well or better than the other model in all cases. In addition, we see that the Braun model performs worse than the other two models when we increase the prior variance of the slope parameters with a larger effect observed with moderate and high correlation. This effect is similar regardless of how the data are generated and, in fact, appears slightly larger when the Braun model is actually the correct model.

Our simulation results suggest that specifying the correct copula model does not improve the operating characteristics of our study compared to simply using an independence model and, in fact, results in worse operating characteristics in many cases. This is counter-intuitive as specifying the correct model should result in more efficient estimates of the model parameters. One possible explanation for this phenomenon is that the correlation parameter between *Y*_*T*_ and *Y*_*E*_ may be too difficult to estimate given the limited sample size. We completed a small simulation study to investigate our ability to estimate *ψ*_1_ and *ψ*_2_ in the Braun and Gumbel copulas, respectively. For each scenario, we considered a study with 11 subjects at each dose level, for a total of 44 subjects, and considered various levels of correlation between *Y*_*T*_ and *Y*_*E*_. 1000 simulations were considered for each scenario. Table 7 presents the mean and standard deviation of the posterior mean for *ψ*_1_ and *ψ*_2_ under various levels of correlation. We see that the Braun model, while certainly biased towards independence, appears to be learning about *ψ*_1_ and has posterior means of approximately 0.65 and 0.80 when *ψ*_1_ is equal to 0.70 and 0.90, respectively. In contrast, there is little information about *ψ*_2_ in the Gumbel model and the posterior means are approximately 0.10 and 0.20 when *ψ*_2_ is equal to 0.40 and 0.80, respectively.

**Table 7:** **Average posterior mean (standard deviation) for the correlation parameters, *ψ***_***k***_**, *k *** **= 1,2, when 11 subjects are treated at each dose level**

**Braun model**	**Scenario 1**	**Scenario 2**	**Scenario 3**	**Scenario 4**	**Scenario 5**
*ψ*_1_=0.5	0.535	0.546	0.531	0.546	0.495
	(0.023)	(0.021)	(0.024)	(0.024)	(0.025)
*ψ*_1_=0.7	0.659	0.668	0.656	0.660	0.643
	(0.020)	(0.018)	(0.021)	(0.019)	(0.021)
*ψ*_1_=0.9	0.784	0.824	0.792	0.777	0.811
	(0.010)	(0.008)	(0.012)	(0.010)	(0.010)
**Gumbel model**	**Scenario 1**	**Scenario 2**	**Scenario 3**	**Scenario 4**	**Scenario 5**
*ψ*_2_=0.0	-0.001	-0.003	0.005	-0.005	-0.002
	(0.075)	(0.081)	(0.067)	(0.067)	(0.072)
*ψ*_2_=0.4	0.115	0.105	0.119	0.091	0.118
	(0.073)	(0.076)	(0.063)	(0.06)	(0.072)
*ψ*_2_=0.8	0.225	0.244	0.194	0.212	0.219
	(0.062)	(0.065)	(0.06)	(0.057)	(0.061)

Data are generated assuming the correct model and specified true correlation between *Y*_*T*_ and *Y*_*E*_ (*ψ*_*k*_).

## Conclusions

We completed a simulation study to evaluate the performance of copula models in phase I-II clinical trials under model misspecification. Our results suggest that the operating characteristics of our study are relatively robust to misspecifying the copula model. Both models exhibited similar performance, as measured by the probability of correctly identifying the optimal dose and the number of subjects treated at the optimal dose, regardless of whether the data were generated from the correct or incorrect copula, even when there is substantial correlation between *Y*_*T*_ and *Y*_*E*_. These results were robust to changes in the maximum sample size and the prior distributions for the parameters of the logistic regression models for toxicity and efficacy. In comparing the two models, there was little difference in the operating characteristics, although, the straight-forward interpretation of the model parameters in the Gumbel model may make the Gumbel model more desirable.

Surprisingly, the naive model that ignores the correlation between *Y*_*E*_ and *Y*_*T*_ performed as well, better in some cases, with respect to correctly identifying the optimal dose and the number of subjects treated at the optimal dose than even the correct model. This was true regardless of the scenario and true correlation between *Y*_*E*_ and *Y*_*T*_. This result is not intuitive as we would expect that correctly specifying the copula model would result in more efficient parameter estimates and improved operating characteristics of our study.

There are several possible explanations for the lack of benefit when utilizing the correct copula model in Phase I-II clinical trials. First, it is possible that the likelihood contains very little information about the correlation parameter and any benefit of modeling the correlation is negated by the need to estimate an additional correlation parameter. In this case, fitting a copula model may result in more variable estimates, in general, which would result in performance that is no better, and potentially worse, than simply assuming that the two endpoints are independent. A second explanation is that Phase I-II clinical trials do not provide sufficient information for selecting the correct copula model. Phase I-II clinical trials utilize small sample sizes, which makes it difficult to properly evaluate the fit of a model. Furthermore, regulatory bodies typically require that a model is specified in advance when utilizing an adaptive trial design. These challenges make it difficult to identify the correct copula from the data, which may negate any benefit from modeling the correlation between the toxicity and efficacy endpoint. Finally, properly modeling the correlation between two endpoints is necessary to complete proper inference (hypothesis tests, credible intervals, etc.) but it may be that modeling this correlation is not necessary in a Phase I-II clinical trial where the goal is to select a dose at study completion regardless of the error associated with estimates of the probability of efficacy and toxicity. In this case, we would not expect any benefit from modeling the correlation, which is consistent with our simulation results.

We completed a second simulation study to investigate the model’s ability to estimate the correlation parameters with the sample sizes used in phase I-II clinical trials in order to fully understand the behavior of copula models in phase I-II clinical trials. Estimates of the correlation parameters were biased towards the prior mean of no correlation in both cases but the average posterior mean of the correlation parameter in the Braun model was much closer to the true value of the correlation parameter than in the Gumbel model. This suggests that, while the likelihood for the Braun model contains a fair amount of information for the correlation parameter, the likelihood for the Gumbel model contains very little information about the correlation parameter and provides a potential explanation for the apparent lack of benefit due to properly modeling the correlation between efficacy and toxicity in phase I-II clinical trials.

The results of this manuscript are dependent the decision rule proposed by Thall and Cook [4]. Other decision rules have been proposed for phase I-II clinical trials [5,13] and it is possible that the results of our simulation study would change with a different decision rule. We think that this is unlikely given that we consistently found no benefit of appropriately modeling the correlation between toxicity and efficacy in all scenarios and additional simulation results illustrated that the likelihood contains little information for estimating the correlation parameters in the two copulas we considered for sample sizes typical of phase I-II clinical trials. Nevertheless, this issue should be considered when evaluating the results of our simulation study.

Our results do not indicate a preference for one model over the other. Both models performed similarly, regardless of how the data were generated. Although, the performance of the Braun model suffered more than the performance of the Gumbel model when vague priors were placed on the slope parameters in the logistic regression models for efficacy and toxicity. The other primary difference between the two models is the interpretation of the model parameters. In the Gumbel model, *π*_*E*_ and *π*_*T*_ represent the marginal probability of efficacy and toxicity, respectively, but are conditional probabilities that depend on the correlation parameter in the Braun model. This property could make the Gumbel model preferable given the similar performance of the two models. That said, our results indicate that it would be acceptable for a practitioner to simply fit the model that assumes independence even though the two outcomes are likely correlated. The performance of the two copula models could possibly be improved by utilizing informative priors for the correlation parameters but strongly informative priors would be required to overcome the apparent lack of information in the likelihood and it is unlikely that such prior information exists in early phase clinical trials. In this case, fitting a model that assumes independence is preferable.

## Competing interests

Both authors declare that they have no competing interests.

## Authors’ contributions

KC implemented the simulation study, interpreted the results and drafted the manuscript. JK conceived of the study and edited the manuscript. Both authors read and approved the final manuscript.

## Pre-publication history

The pre-publication history for this paper can be accessed here:

http://www.biomedcentral.com/1471-2288/14/51/prepub
